# The Indirect Bonding Technique in Orthodontics—A Narrative Literature Review

**DOI:** 10.3390/ma13040986

**Published:** 2020-02-22

**Authors:** Agnieszka Nawrocka, Monika Lukomska-Szymanska

**Affiliations:** Department of General Dentistry, Medical University of Lodz, Pomorska 251, 92-213 Lodz, Poland; agnieszka.nawrocka@stud.umed.lodz.pl

**Keywords:** indirect-bonding progress, digital technologies, orthodontics

## Abstract

The technique described as indirect bonding is an alternative to the conventional intraoral method of bracket placement. The appliance position is planned and fixed on a plaster model and then transferred into the oral cavity. Indirect bonding is a precise and time-saving technique of bracket placement, growing in popularity in recent years. It provides a combination of great precision with time efficiency. The fundaments of the indirect bonding technique are presented here. From the first clinical trial conducted almost fifty years ago, the method has evolved; the progress that has been made is described. Modern technologies involving computer scanning and manufacturing have led to great precision in bracket placement. Digital innovations such as rapid prototyping and stereolithography open up a new avenue of research and represent the next steps in indirect technique development. Individual 3D transfers are convenient in difficult clinical cases and can improve the effectiveness of the procedure, reduce the number of technical stages and reduce total chairside time. This paper also summarizes the advancement in adhesive materials, including an overview of advantages and disadvantages of different types of bonding resins and of the mean shear bond strength (SBS) achieved in the indirect procedure.

## 1. Introduction

Proper bracket placement is crucial in orthodontic treatment and with a suitable arch wire provides the desired mechanical effect. Imprecision in bracket location may lead to unwanted tooth movement: unplanned torque, rotation and trusion. The introduction of adhesion was a revolution in orthodontics and facilitated appliance fixing procedures. There are two main techniques of bracket placement. The first one, more popular, is direct bonding: the braces are adjusted directly on patients’ teeth. The second technique is called indirect bonding. The appliance position is planned and fixed on a plaster model and then transferred into the oral cavity. Indirect bonding is a precise and time-saving method of bracket placement, growing in popularity in recent years.

The aim of this paper is to present the development of the indirect bonding technique, on the basis of the current literature. The method undergoes constant evolution and the progress that has been made is the subject of the present article. In addition, the purpose is to provide a comparison of adhesive materials used in indirect bonding protocols, including comparing types of polymerization (chemically cured, thermally cured and light-cured resins) and shear bond strength (SBS) values.

## 2. The Beginnings of the Indirect Technique (1970–1999)

The innovative indirect bonding technique was developed in 1972 by Silvermann [1] in response to ongoing progress in adhesive techniques, and aimed to improve the precision of bracket placement with a limited amount of chairside time. Although adhesion in orthodontics was introduced in the 1960s, the procedure of bonding had many limitations [2]. Epoxy adhesives used in the pioneering study had prolonged curing times. It required 15–30 min to achieve a gelling phase and maintain the bracket in a proper position. Due to the fact that final hardness was only reached after four days, it was impossible to insert an arch wire and apply the force in one visit [1].

**Figure 1:**
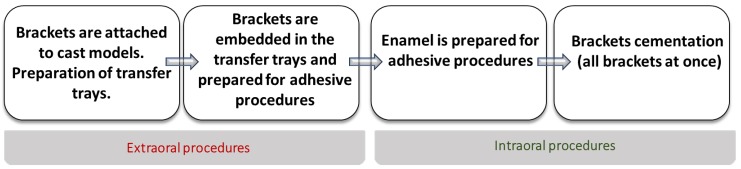
The stages of Silvermann’s technique.

Extraoral bracket adjustment served as a foundation of Silvermann’s idea [3]. Firstly, the whole set of metal orthodontic attachments was placed on the cast model in a desirable location. With one drop of cement, each bracket was fixed to the model in exactly the position that would be maintained in the oral cavity. The Vanguard unit (a vacuum formed tray) was used as a transfer. The adhesive techniques made it possible to fix the brackets intraorally. Hence, after the gentle removal of the tray, each bracket remained attached to the tooth surface. The technique is presented step-by-step in Figure 1.

From the first demonstration of the indirect bonding technique in the 1970s, adhesive procedures continued to develop. However, the basic principles of Silvermann’s method remain valid. New generations of bonding agents are in use, and modern digital technologies are widely applied in orthodontic practice. These include cone-beam computer tomography (CBCT) and Computer Aided Design/Computer Aided Manufacturing (CAD/CAM). Still, extraoral bracket placement followed by transfer to the intraoral environment remains unchanged.

In 1979, research was conducted to improve the effectiveness of the indirect bonding technique, resulting in the development of custom-made bracket bases. In Thomas’ method, brackets were attached to the previously prepared model with chemically cured composite [4,5]. Consequently, a precise bonding pad, including the bracket with the composite resin, was created for each tooth. In the next step, a transfer was used to reproduce the position of the brackets intraorally and the appliance was attached on the teeth using the two-part unfilled resin. The first thin layer of adhesive covered the bracket pads. The second component of resin, a sealant, was spread on the enamel surface. Adhesion between tooth and orthodontic bracket was obtained as a consequence of chemical reaction. The method was precise, but insufficient bond strength was observed. Moreover, the transfer tray was not transparent, and the optimal amount of cement was hard to achieve. These shortcomings triggered further investigation [5].

Clinical trials conducted in the 1980s with heat-cured resins were not satisfying. Brackets did not stay firmly on the surface during heating of the model. Moreover, the method was not suitable for ceramic appliances due to the fact that the model was heated to temperatures exceeding 350 °C for 30 min [6].

Over the years, with the development of dentistry, new generations of bonding agents and composite resins were implemented. Not only in restorative procedures, but also in orthodontics, light-cured cements and sealants came into use [3,5,6,7,8]. Differences in bond strengths according to type of composite became a subject of research [5,8,9,10,11,12,13,14] (Table 1 and Table 2). The comparison of results is possible due to the consistent methodology of SBS testing. Research was conducted in vitro on extracted teeth (human or bovine) and the samples were randomly divided into groups depending on the adhesive material applied. The direct bonding procedure serves as a gold standard and most studies included it as a control group. The sample preparation (cleaning, embedding in acrylic base, impressions, cast and silicone transfer tray preparation) was standardized, well-established and repeatable. After final attachment of brackets to the enamel surface and material setting, a debonding procedure with SBS testing was implemented. Researchers commonly used a universal testing machine to generate a shear load. The tip of the device shifted parallel to the long axis of the tooth as close as possible to the bracket-enamel connection [5,8,9,10,11,12,13,14].

According to the polymerization method, materials used for indirect bonding were classified as chemically cured, thermally cured or light-cured resins. The type of polymerization was a factor determining the setting and working time. As a consequence, it had an influence on the formation of the custom base, on the precision of bracket placement and on achieved SBS (Shear Bond Strength). In this research, Thomas’ original technique was modified by changing the type of adhesive applied. Debonding procedures performed with a universal testing machine revealed differences in SBS depending on the applied material. Thermally cured composites showed significantly higher probability of bonding failure. Brackets cemented with chemically cured and light-cured composites displayed the highest bond strengths, similar to the results achieved in direct techniques. SBS values reached 15.41 MPa and 14.99 MPa [5]. According to Reynolds, these values are sufficient for orthodontic purposes: Reynolds suggested that minimum bonding strengths should range between 5.9 MPa and 7.8 MPa [11].

In 1991, the system of precoated brackets was introduced as another time-saving improvement [12]. Laboratory research and clinical trials did not reveal significant differences in failure rates between precoated and noncoated elements [13,14].

## 3. Development of Bonding Systems

**Figure 2:**
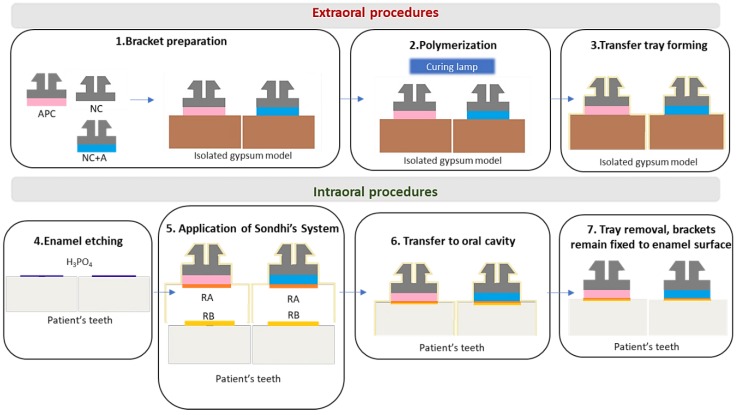
Sondhi’s indirect bonding procedure. APC—Adhesive Precoated Bracket; NC—Non-coated bracket; A—orthodontic adhesive; RA—resin A in Sondhi’s System; RB—resin B in Sondhi’s System.

The next step in the indirect technique was proposed in 1999 by Sondhi [6,15]. The main aim was to introduce a new bonding system specifically designed for extraoral bracket placement. It was noticed that the bonding system used in direct techniques was not compatible with indirect methods. The major problem was the working time. In conventional intraoral bonding, the working time should be longer to enable the clinician to fix the bracket in a proper position, whereas a prolonged curing process is not necessary after placing the transfer tray. Sondhi introduced a new resin with increased viscosity, specifically designed for the indirect technique. With the addition of 5% silica filler, the bond had an improved ability to fill the imperfections on the bracket base or enamel surface. Another advantage was the optimal setting time. Just 30 s after placing the trays in a patient’s mouth, they did not need to be held by the operator, and in 2 min they could be safely removed from the oral cavity. Two types of brackets—precoated (APC = Adhesive Precoated Brackets) and non-coated—were tested [6,15]. The non-coated brackets were covered with TransbondXT LightCure Adhesive to form a custom adhesive base. The procedure proposed by Sondhi is presented in Figure 2.

**Figure 3:**
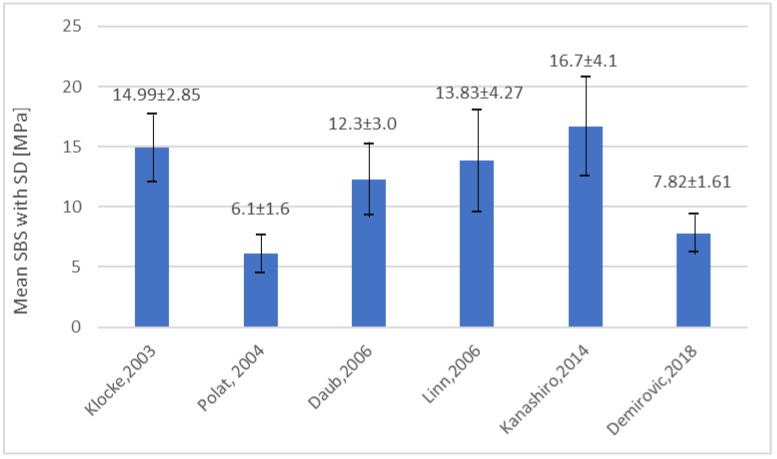
SBS of Sondhi’s Rapid Set in different articles.

The specially designed bonding system led to promising outcomes. The first important advantage was satisfactory initial bond strength. It was crucial to apply the orthodontic force. The bond strength after 24 h reached values not diverging from other resins. According to one in vitro study, application of Sondhi’s system with chemically cured sealant ensured that the mean value of bond strength reached 14.99 MPa [5]. This was higher than the gold standard, direct bonding with light-cured composite, which reached a mean value of 13.88 MPa. However, further research did not prove the advantage of Sondhi’s resin (Figure 3) [10,16,17].

Since orthodontic brackets transfer force to teeth, adequate bond strength is required to provide proper and precise fixation [17]. Results of SBS revealed no statistical and clinical differences between the values obtained in the direct and indirect techniques (Table 1) [3,5,7,8,9,17]. Moreover, bonding failure rates, total chairside time and the number of visits were comparable [18]. However, when adhesive remnant index (ARI) was assessed in a different study, bonding failure in the indirect technique was most prevalent in the area between the bracket base and the tooth surface [16].

It was estimated that 2–6% of brackets bonded indirectly revealed an undesirable loss, probably due to insufficient connection with dental hard tissues [19]. To decrease the risk of unwanted debonding of custom-based brackets, researchers suggested improving the properties of bonding resin by adding an “adhesion booster”. This chemical agent contained a composition of biphenyl dimethacrylate and hydroxyethyl methacrylate. As a result, the bonding resin gained hydrophilic groups and its ability to penetrate the etched enamel was improved. SBS was significantly higher, because the adhesion booster could compensate for imperfections responsible for increasing the risk of failure, such as aprismatic or hypomineralized enamel structure [19].

## 4. The 21st Century—Time for Digital Technology

The 21st century is the age of modern digital technologies in orthodontic practice. To improve precision of bracket placement, several computer-aided solutions have been proposed. A new approach, presented in 2006, connects the indirect bonding method with CAD/CAM technology [20]. The method, described as rapid prototyping, is used to prepare precisely designed transfer trays. At first, the traditional impression with the silicon base material is taken. The obtained model is then scanned with a high resolution 3D scanner to achieve a digital record. The corresponding software enables clinicians to design the bracket placement virtually with great accuracy (0.1 mm). Next, the 3D individual transfers—the rapid prototyping trays (RPT) for each tooth—are printed. In the following step, braces are fixed intraorally. Teeth are prepared according to adhesive bonding guidelines: etching with 37% orthophosphoric acid, rinsing, drying and applying a thin layer of primer. The bases of orthodontic brackets are covered with the adhesive material and then inserted carefully in their determined location in RPT. The trays with brackets are placed on the teeth and after the light-induced polymerization process the transfer (RPT) is removed, while brackets remained attached to the enamel surface [20].

**Figure 4:**
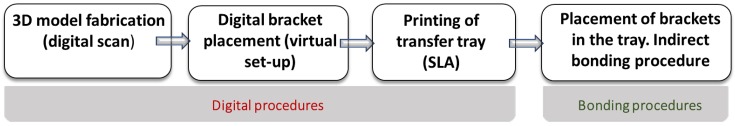
The virtual orthodontic technique.

In 2011, another virtual orthodontic technique was introduced [21]. It should be emphasized that authors performed 3D digital set-up to improve bracket placement. Stereolithography—a type of rapid prototyping method, also known as optical fabrication technology—was used to create transfer trays described also as “jigs” (Figure 4).

The accuracy of CAD/CAM-aided bracket placement has been questioned [22]. When traditional gypsum casts are compared with computer imaging, the digital technique is considered less precise in mapping the tips of dental cusps. From an orthodontic point of view, the tip of the dental cusp is an important point of reference for determining the bracket position. Attrited teeth with shorter cusps revealed more errors in digital projection [22]. However, the research proved that although more inaccuracies may occur compared with the traditional technique, these minor deficiencies can be eliminated with dedicated software and virtual set-ups [23]. The authors confirmed that CAD/CAM technology provides accurate bracket placement in laboratory conditions [23]. The issue was to assess if the ideal transfer of digital project is also achievable in the chairside environment. A “human factor”—the orthodontist—was blamed for slight changes in the computer-defined bracket location. In particular, lower reproducibility was expected in difficult cases of crowding with rotated and tipped teeth. However, assessment with cone-beam computer tomography (CBCT) supported with 3D digital models did not confirm this hypothesis. Orthodontists strictly followed pre-determined bracket position. When the ideal location was chosen and planned with the CAD/CAM technique, it was kept even several weeks after the beginning of the therapy [23,24,25].

Another modality of the indirect bonding technique was introduced in 2015. The method is based on the Bracket–Archwire Assembly (BAA) and does not require transferring trays and digital technology. A large, heavy stainless steel arch wire (for instance, 19” × 25”) with attached brackets is fixed in molar band tubes as a template. The author recommended this technique in the course of treatment of surgical cases [26].

The procedure of indirect bonding also has a practical use in fixing mandibular retainers [27]. In the post treatment stage, it is essential to provide proper retention and stabilize the orthodontic treatment outcome. The indirect technique was proven to be faster in clinical practice than bonding the appliance directly in the oral cavity. Although there was no significant difference in the failure rates of direct and indirect bonded retainers, the latter seem to generate fewer post treatment complications [27,28].

## 5. Conclusions

Developments in indirect bonding have made the technique easier to introduce in clinical practice. The main advantage is improved accuracy with limited chairside time. Indirect bonding is especially indicated in severe malocclusion with crowding and rotations. In these cases, the proper bracket placement is very hard to achieve with direct techniques. Indirect bonding aided with digital technologies provides better, easier and more precise bracket location. Therefore, modern technologies seem to be a favourable solution facilitating orthodontic treatment and providing promising results.

## Figures and Tables

**Table 1:** Mean shear bond strength (SBS) of different materials used in the indirect bonding technique.

Author (Year)	Data from Research				
	Method	Material	Number of Teeth	Mean SBS (SD)	Conclusions
Hocevar et al., 1988 [7]	Direct	Concise (3M, St Paul, Minn, USA)	18	6.69 (0.92)	NSD
	Indirect	Concise (3M, St Paul, Minn, USA)	18	6.40 (0.87)	
Milne et al., 1989 [9]	Direct	Concise (3M, St Paul, Minn, USA)	12	19.06 (1.67)	NSD
	Indirect	Concise (3M, St Paul, Minn, USA)	12	16.77 (3.04)	
Yi et al., 2003 [3]	Direct	Transbond XT (3M Unitek, Monrovia, CA, USA)	27	10.9 (2.9)	NSD
	Indirect	APC, Sondhi Rapid Set (3M Unitek, Monrovia, CA, USA)	27	11.2 (2.6)	
Klocke et al., 2003 [5]	Direct	Transbond XT (3M Unitek, Monrovia, CA, USA)	20	13.88 (4.33)	TC: lower SBS LC, CC: NSD
	Indirect	Thermacure+MaximumCure (Reliance Orthodontic Products, Itasca, Ill, USA)	20	7.28 (4.88)	
		Thermacure+Custom IQ (Reliance Orthodontic Products, Itasca, Ill, USA)	20	7.07 (4.11)	
		PhaseII+MaximumCure (Reliance Orthodontic Products, Itasca, Ill, USA)	20	15.41 (3.21)	
		APC, Sondhi Rapid Set (3M Unitek, Monrovia, CA, USA)	20	14.99 (2.85)	
Polat et al., 2004 [10]	Direct	Transbond XT (3M Unitek, Monrovia, CA, USA)	20	12,8 (5.4)	Transbond XT + SondhiRapidSet: lower SBS
	Indirect	Thermacure+CustomIQ+adhesion booster (Reliance Orthodontic Products, Itasca, Ill, USA)	20	10.3 (4.1)	
		Transbond XT, Sondhi Rapid Set (3M Unitek, Monrovia, CA, USA)	20	6.1 (1.6)	
Linn et al., 2006 [8]	Direct	Transbond XT (3M Unitek, Monrovia, CA, USA)	20	16,27 (4.74)	NSD
	Indirect	Transbond XT, Sondhi Rapid Set (3M Unitek, Monrovia, CA, USA)	20	14.76 (4.06)	
		Enlight LV, OrthoSolo (Orm- co Corporation, Glendora, Calif, USA)	20	13,83 (4.27)	

**Legend**: TC—Thermally Cured Material; CC—Chemo-Cured Material; LC—Light-Cured Material; SBS—Shear Bond Strength; SD—Standard Deviation; NSD—No Significant Difference

**Table 2:** Advantages and disadvantages of various types of materials used in the indirect bonding technique [4,5,8,15,16,17].

Enamel Preparation	Adhesive on Bracket Base	Sealant	Advantages	Disadvantages
**AET**	CC	CC(2 components, (catalyst on bracket base and universal unfilled resin on enamel)	-Less failures than in Silvermann’s technique [5] -Better control of the material amount [4]	-Limited time for bracket placement [8,15] -Incomplete curing of sealant [5,8]
**AET**	TC	CC(2 components, mixed before application)	-Unlimited time for bracket placement [8] -Reduced risk of material excess [8]	-Lower SBS in comparison to LC and CC bracket bases [5,16] -Not recommended for ceramic brackets [16]
**AET**	LC	CC (2 components, mixed before application)	-Unlimited time for bracket placement [8] -Fast polymerization [8,15] -Reduced risk of material excess [5,8] - Bond strengths similar to the DB [15]	The risk of bonding failure between sealant and composite [5]
**SET**	LC	SET, LC (2 components, mixed before an application)	Reduced amount of adhesive after debonding (lower ARI) in comparison to AET [17]	Lower SBS than AET [17]

**Legend:** TC—thermally cured material; CC—chemo-cured material; LC—light-cured material; SBS—shear bond strength; SET—self-etch technique; AET—acid-etch technique; DB—direct bonding
